# Evidence of Egg Diversity in Squamate Evolution from Cretaceous Anguimorph Embryos

**DOI:** 10.1371/journal.pone.0128610

**Published:** 2015-07-15

**Authors:** Vincent Fernandez, Eric Buffetaut, Varavudh Suteethorn, Jean-Claude Rage, Paul Tafforeau, Martin Kundrát

**Affiliations:** 1 European Synchrotron Radiation Facility, 71 rue des Martyrs, 38043, Grenoble, France; 2 Evolutionary Studies Institute, University of the Witwatersrand, Wits 2050, Johannesburg, South Africa; 3 CNRS (Centre National de la Recherche Scientifique) UMR (Unité Mixte de Recherche) 8538, Laboratoire de Géologie de l’Ecole Normale Supérieure, 24, Rue Lhomond, 75231, Paris, France; 4 Palaeontological Research and Education Centre, Mahasarakham University, Khamrieng Sub-district, Kantharawichai District, Maha Sarakham Province, 44150, Thailand; 5 Sorbonne Universités—CR2P - MNHN, CNRS, UPMC-Paris6, Muséum national d’Histoire Naturelle, 57 rue Buffon, CP 38, 75005, Paris, France; 6 Subdepartment of Development and Evolution, Department of Organismal Biology, Evolutionary Biology Centre, Uppsala University, Norbyvägen 18A, 752 36, Uppsala, Sweden; 7 Department of Biomathematics, Institute of Physiology, Academy of Sciences of the Czech Republic, Vídeňská 1083, 142 20, Praha, Czech Republic; University of Pennsylvania, UNITED STATES

## Abstract

Lizards are remarkable amongst amniotes, for they display a unique mosaic of reproduction modes ranging from egg-laying to live-bearing. Within this patchwork, geckoes are believed to represent the only group to ever have produced fully calcified rigid-shelled eggs, contrasting with the ubiquitous parchment shelled-eggs observed in other lineages. However, this hypothesis relies only on observations of modern taxa and fossilised gecko-like eggshells which have never been found in association with any embryonic or parental remains. We report here the first attested fossil eggs of lizards from the Early Cretaceous of Thailand, combining hard eggshells with exquisitely preserved embryos of anguimoph (e.g. Komodo dragons, mosasaurs). These fossils shed light on an apparently rare reproduction strategy of squamates, demonstrate that the evolution of rigid-shelled eggs are not an exclusive specialization of geckoes, and suggest a high plasticity in the reproductive organs mineralizing eggshells.

## Introduction

**Fig 1:**
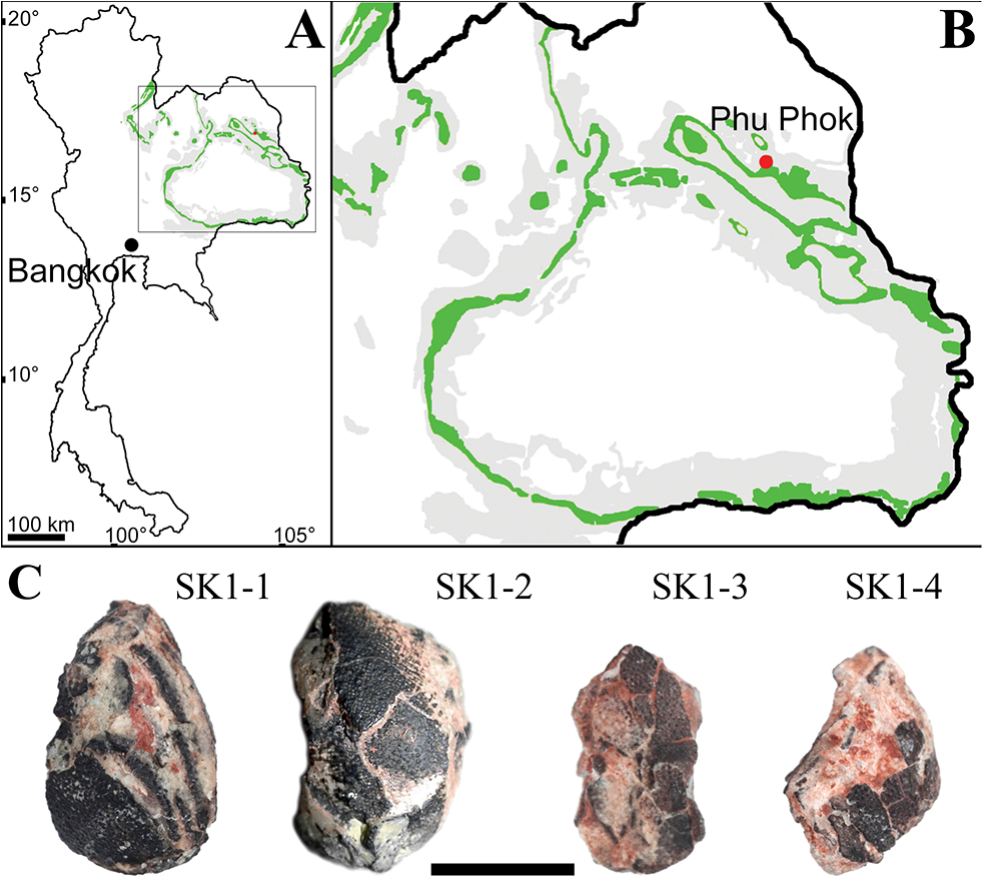
Material and geological settings. **A**, map of Thailand showing outcrops of the Sao Khua Formation (in green) and **B**, close-up on north-eastern-Thailand with location of Phu Phok; **C**, and photograph of 4 of the eggs from Phu Phok (SK1-1, SK1-2, SK1-3 and SK1-4). Scale bar, 1 cm.

**Fig 2:**
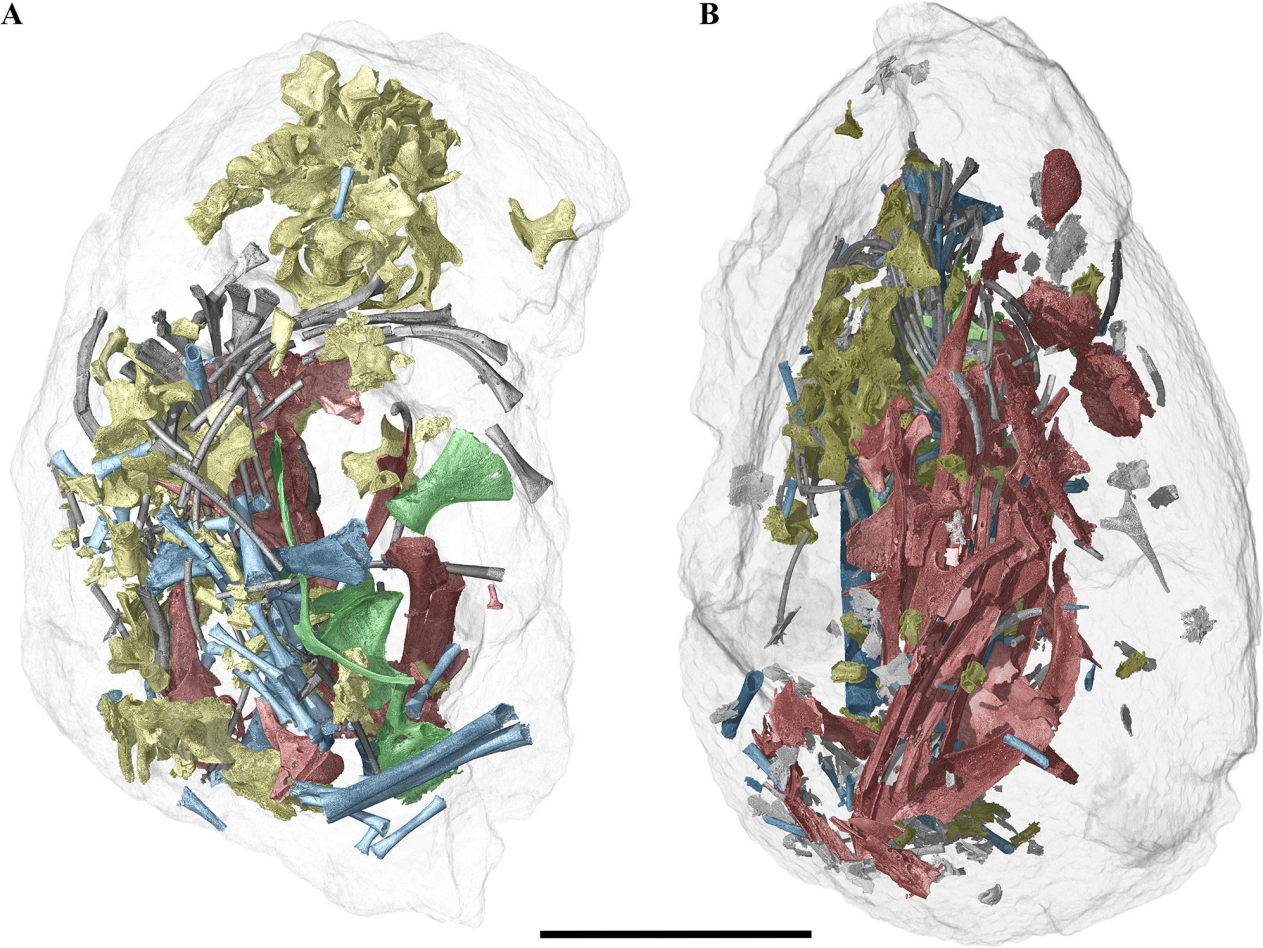
Three-dimensional rendering of two fossil eggs and their enclosed embryonic bones from Phu Phok. **A**, SK1-2. **B**, SK1-1. Colours: red, skull and mandible; yellow, vertebrae; grey, ribs; green, pectoral and pelvic girdle; blue, limbs. Scale bar, 5 mm.

Fossilised eggshells attributed to squamates have been reported from localities covering the Early Cretaceous to the Early Miocene [1–6]. Due to similarities with the eggs of modern gekkonids, the only squamate capable of producing fully calcified and rigid-shelled eggs [5,7–9], these eggshells have been tentatively assigned to the Gekkota [1,2,4,6]. The lack of embryonic or parental indicators associated with these fossilised eggshells casts doubt on the reliability of such conclusions and hence, to their application for our understanding of the evolution of reproductive strategies in oviparous squamates. The re-interpretation of the minute fossil eggs from the Barremian [10] of Phu Phok (north-eastern Thailand, Fig 1) provided the first reliable material to address these questions. The use of propagation phase contrast synchrotron microtomography on these eggs (Fig 2 and S1 Fig) previously attributed to Theropoda [11], resulted in the striking discovery of anguimorph embryos within rigid-shelled eggs, which were thought to be an exclusive evolutionary specialisation of gekkonid lizards [11].

## Material and Geological Setting

The fossilized eggs presented here were surface collected by an international team led by one of us (V.S), from red siltstones of the Sao Khua Formation at the locality of Phu Phok (SK1), Sakhon Nakhorn Province, north-eastern Thailand (Fig 1). In total seven eggs have been discovered from this locality during the course of different official field campaigns of the Royal Thai Department of Mineral Resources (DMR): five eggs were discovered in 2002 and 2003 (specimen SK1-1 to SK1-5) [11]; specimen SK1-6 and SK1-7 were discovered in 2007 and 2008 respectively. As it was poorly preserved, specimen SK1-5 was thin sectioned for characterization of the eggshell [11]. No nesting structure was observable although the eggs were scattered in the sediment over a relatively small area (about 2 m^2^). No permits were necessary. The DMR is a governmental organisation which has permission to do fieldwork upon acceptance of the land owner. The locality of Phu Phok belongs to the Thai government and therefore no permit was necessary for prospection and collection at the site. While the current legislation stipulates the necessity of permits to transport fossils out of Thailand, it was not the case at the time the fossils were collected (in 2005). Since then, the fossils have been returned to the collection of the Sirindhorn Museum in Phu Kum Khao (Sahatsakhan District, Kalasin Province, Thailand). Therefore no permits were necessary for prospection or for transportation which complied with all relevant regulations.

The Sao Khua formation is mainly characterised by floodplain deposits including sandstone, siltstone and mudstone, together with common calcretes which reflects a low-energy fluvial environment. The Sao Khua Formation is part of the Khorat Group, the latter consisting of a series of five non-marine formations deposited in a thermal sag basin during the Late Jurassic-Early Cretaceous [12]. The accompanying fauna includes fishes, turtles, crocodilians and dinosaurs. Palynological evidence suggested a Berriasian-Barremian age for the Sao Khua Formation [13]. A late Barremian age is indicated by freshwater bivalves [10]. While the fauna from the older Phu Kradung Formation and the younger Khok Kruat Formation show some resemblance with their contemporaneous counterparts from Asia, the peculiar fauna of the Sao Khua Formation suggests that the Khorat region was somehow isolated from the main Eurasian continent [14].

## Methods

### Characterization of entire eggs

**Fig 3:**
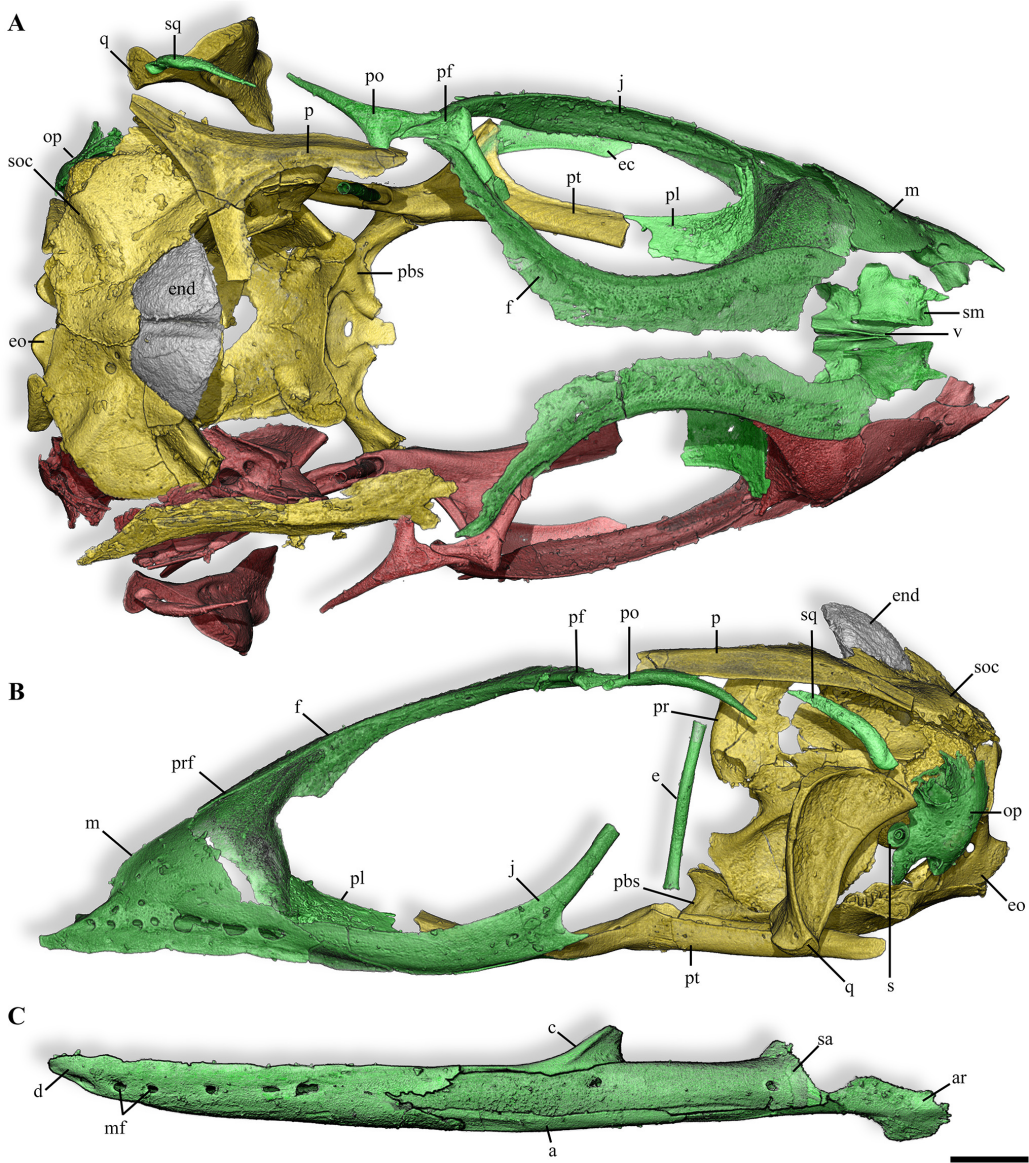
Skull and mandible of the anguimorph embryos from Phu Phok. **A,B,** skull, dorsal (A) and lateral (B) views. **C**, left mandible, lateral view. Colours: yellow, SK1-1; green, SK1-2; red, absent or incomplete bone replaced by symmetrical reconstruction. Anatomical abbreviations: a, angular; ar, articular; c, coronoid; d, dentary; e, epipterygoid; ec, ectopterygoid; end, calcified endolymph; eo, exoccipital; f, frontal; j, jugal; m, maxilla; mf, mental foramen; op, opisthotic; p, parietal; pbs, parabasisphenoid; pf, postfrontal; pl, palatine; po, postorbital; pr, prootic; prf, prefrontal; pt, pterygoid; q, quadrate; s, stapes; sa, surangular; sm, septomaxilla; soc, supraoccipital; sq, squamosal; v, vomer. Scale bars, 1 mm.

**Fig 4:**
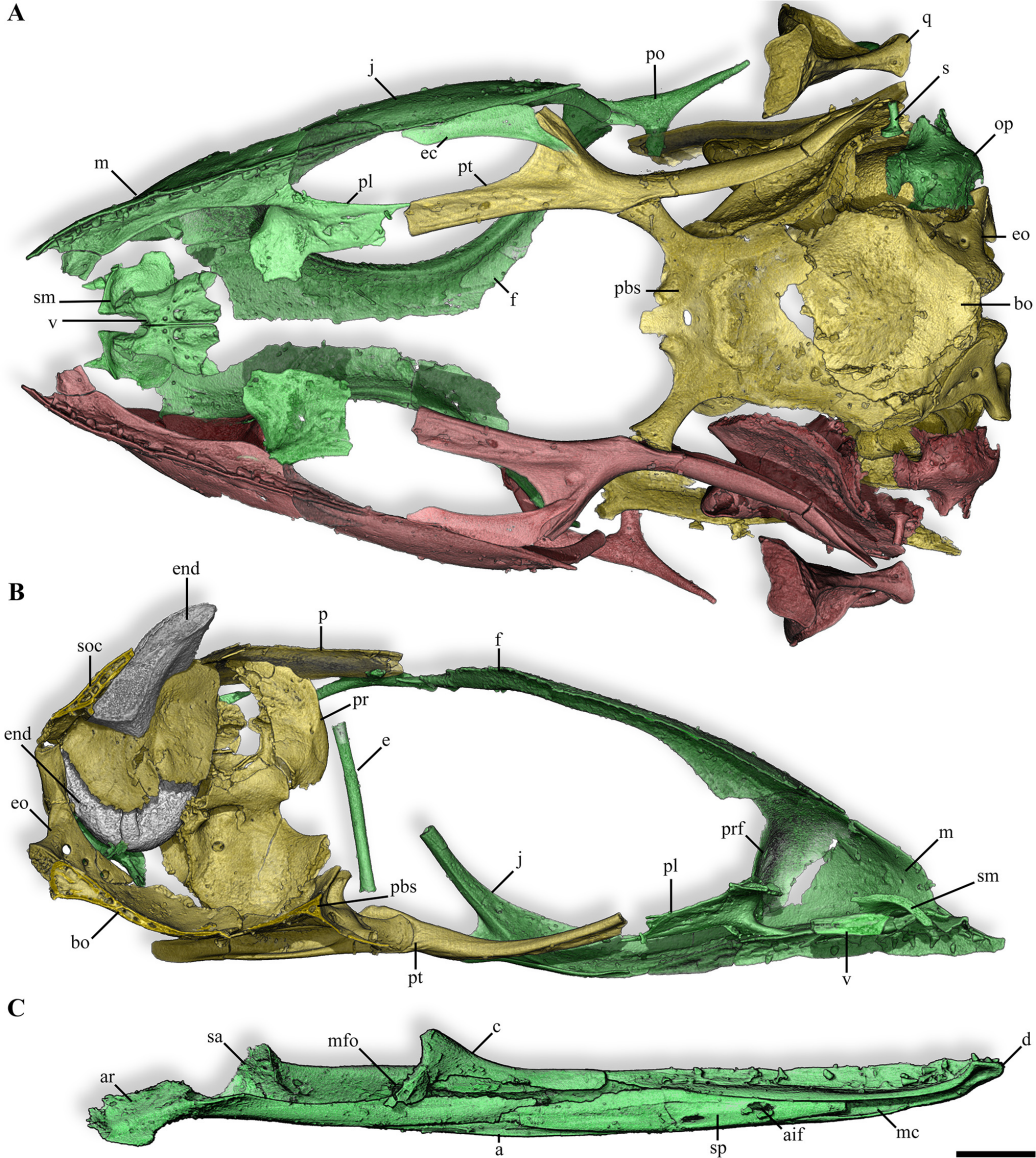
Skull and mandible of the anguimorph embryos from Phu Phok. **A, B,** skull, ventral (A) and medial view of a sagittal section (B) views. **C**, left mandible, medial view. Colours: yellow, SK1-1; green, SK1-2; red, absent or incomplete bone replaced by symmetrical reconstruction. Anatomical abbreviations: a, angular; aif, anterior inferior alveolar foramen; ar, articular; bo, basioccipital; c, coronoid; d, dentary; e, epipterygoid; ec, ectopterygoid; end, calcified endolymph; eo, exoccipital; f, frontal; j, jugal; m, maxilla; mc, Meckelian canal; mfo, mandibular fossa; op, opisthotic; p, parietal; pbs, parabasisphenoid; pl, palatine; po, postorbital; pr, prootic; prf, prefrontal; pt, pterygoid; q, quadrate; s, stapes; sa, surangular; sm, septomaxilla; soc, supraoccipital; sp, splenial; v, vomer. Scale bars, 1 mm.

**Fig 5:**
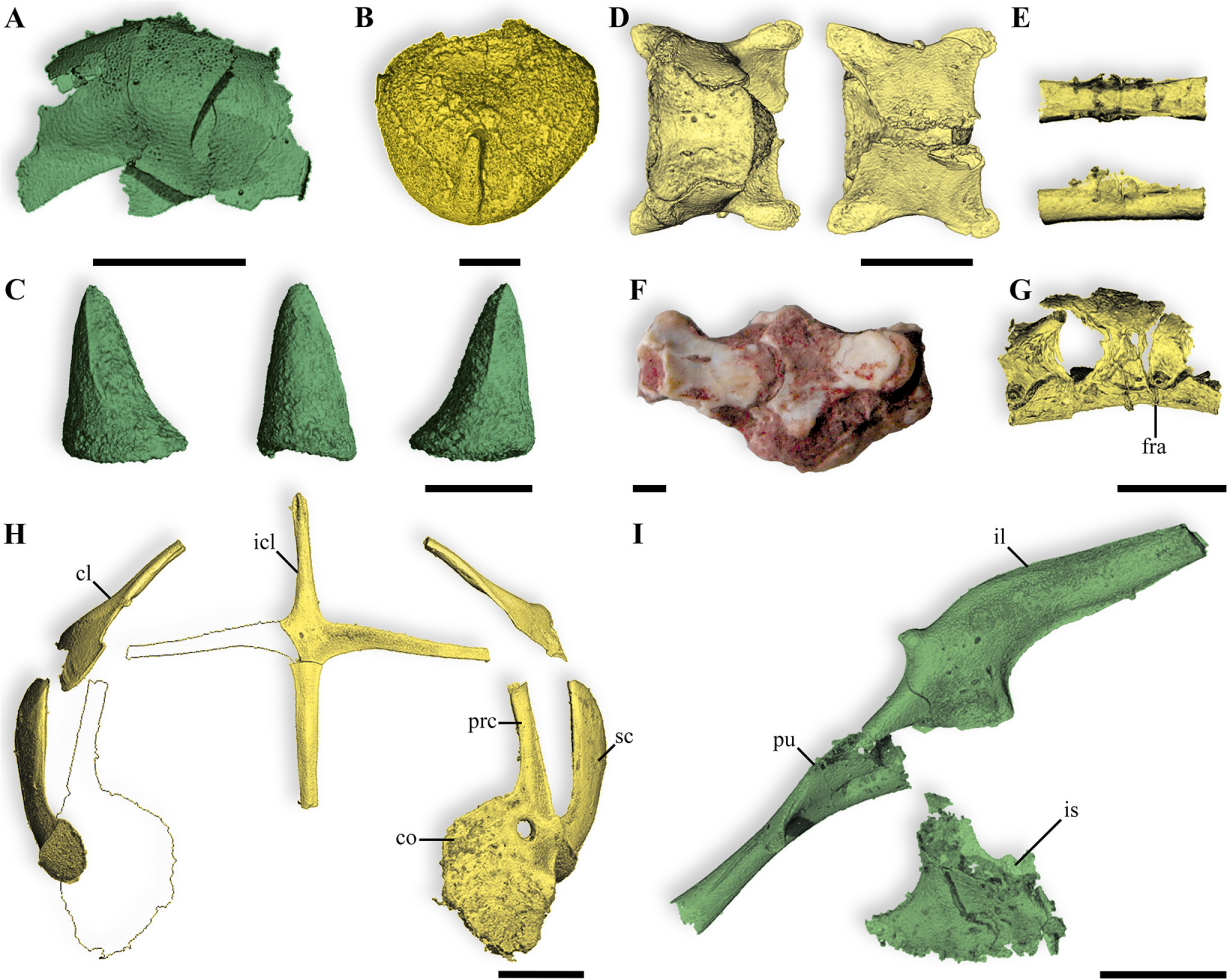
Anguimorph cranial and post-cranial material from Phu Phok. **A**, segments of the sclerotic ring, lateral view. **B**, Calcified endolymph from the left sacculus. **C**, tooth from the anterior part of the dentary in cranial, labial and rostral views; **D**, presacral vertebra, ventral and dorsal views. **E**, caudal vertebral centrum, dorsal and lateral views. **F**, two consecutive presacral vertebrae from Phu Phok, attributed to an anguimorph, ventral view. **G**, caudal vertebrae, near the sacral region, lateral view. **H**, reconstruction of part of the pectoral girdle, ventral view. **I**, reconstruction of the pelvic girdle, lateral view. Colours: yellow, SK1-1; green, SK1-2; red, absent or incomplete bone replaced by symmetrical reconstruction. Anatomical abbreviations: cl, clavicle; co, coracoid; fra, autotomous fracture plane; icl, interclavicle; il, ilium; is, ischium; prc, procoracoid; pu, pubes; sc, scapula. Scale bars, 1 mm.

**Fig 6:**
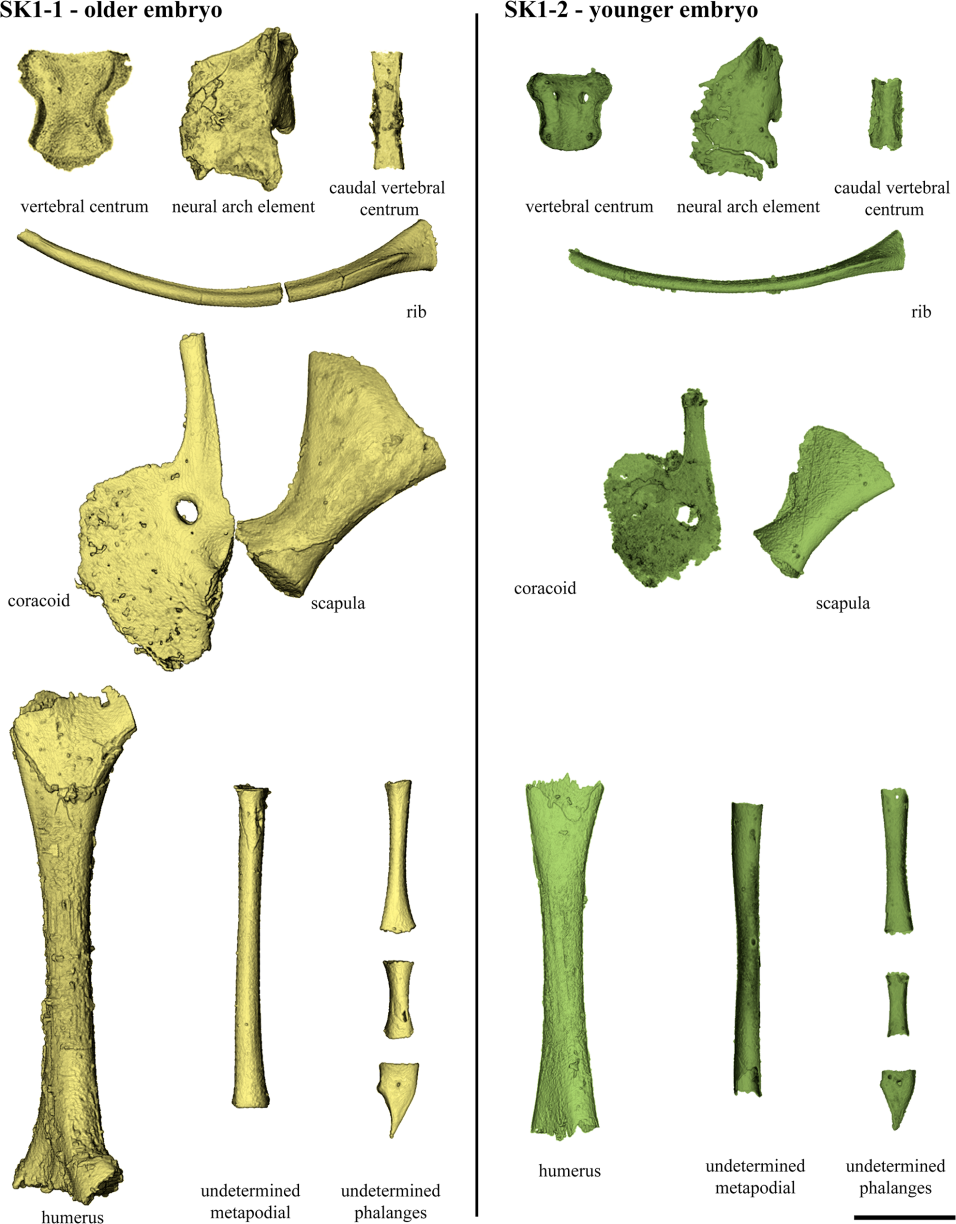
Comparison of the ossification extension of several postcranial components from the embryos SK1-1 and SK1-2. The vertebral elements compared (both pre- and post-sacral), as well as the rib, are the largest ones from the eggs SK1-1 and SK1-2. In the pectoral girdle, SK1-1 shows an advance degree of ossification notably in the extension of the procoracoid, the ventral margin of the glenoid fossa and the blade of the scapula. Ossification toward epiphyses of the humerus is more advanced in SK1-1. Scale bar, 1 mm.

The eggs SK1-1, SK1-2, SK1-3, SK1-4, SK1-6 and SK1-7 were scanned at the ID19 beamline of the European Synchrotron Radiation Facility (ESRF). They were scanned following a propagation phase contrast protocol (sample/detector distance of 990 mm) associated to a 51 keV monochromatic beam (double Si 111 monochromator) and a half-acquisition protocol [15]. The tomography was computed based on 3600 projections (2048 x 2048 pixels) of 2.5 seconds each over 360 degrees with a 5.06 microns isotropic voxel size. As the vertical field of view could not cover the full height of an egg, multiple scans were necessary for each specimen. The reconstructed volumes were stitched together to visualize the whole eggs and by optimizing the overall contrast (i.e. stretching the range of grey values from the 32 bit raw data into a 8 bit full range of values, avoiding too high levels of saturation). The three dimensional processing was performed using VG Studio MAX 2.1 (Volume Graphics, Heidelberg, Germany) following the segmentation protocol described in previous study [16]. Reconstruction of the skeleton from the 3D rendering was done first using bones from the oldest embryos (SK1-1, yellow bones in Figs 3–6) and completed by bones from the youngest embryo (SK1-2, green bones in Figs 3–6); Missing and incomplete bones were replaced by symmetrical rendering of their counterpart (red bones in Figs 3 and 4). Finally, cranial and post-cranial bones were printed in 3D using a Dimension Elite fused filament fabrication prototyper (Statasys, Eden Prairie, U.S.A), and used for our anatomic investigation as well as for a composite reconstruction. The 3D prototypes of most of the bones helped to visualize and describe their morphology but also to study the contacts between them. Based on the observation of the contact using the 3D prototypes, we produced a 3D rendering reconstruction of the skull, the mandible, the pectoral and pelvic girdle and a few vertebrae (Fig 2 and S3 Fig). The data reported in this paper are archived at the following database http://paleo.esrf.eu/.

Measurements of the eggshells were made using the tomographic reconstructions and the calliper tool of VGstudiomax 2.2. For each measure, the structure was properly oriented in the tree orthogonal views, insuring the validity of the value. As the voxel size of the tomographic data is 5.06 microns, the measures are considered valid up to 2 pixels (10.12 microns). Measures were only taken on shell fragments that had slid inside the egg, to avoid potential structure damaged by weathering and diagenesis.

### Characterization of thin section (egg SK1-5)

Thin sections of the eggshell were studied using transmitted light microscopy, X-ray radiography and X-ray diffraction.

For transmitted light microscopy, we used a Leica DMR XP microscope. Images were recorded with Leica DC 300 digital camera and the software Photostudio 5.5 (Arcsoft, Fremont, USA). Each frame was recorded twice, using polarized transmitted light and analysed polarized transmitted light.

The X-ray radiography of the thin section was performed at the ID19 beamline of the ESRF, using a 19.6 keV pink beam, a sCMOS pco.edge 5.5 camera, and an optic setup producing radiographs with an isotropic pixel size of 0.7 microns. Each radiograph was obtained from 1.2 seconds of exposure. The mesh of radiographs was performed at a sample-detector distance of 200 mm to observe phase contrast edge enhancement effect. We performed single-distance phase retrieval on the radiographs using the ANKA phase plug-in [17] of ImageJ [18]. The delta-beta value was set to 169. The radiographs obtain by phase-retrieval were stitched together using the Grid/Collection Stitching plug-in [19] of Fiji [20].

The diffraction experiment was performed on the ID19 beamline of the ESRF. We used a focused beam (Be lenses) and bended double Laue crystals to produced a monochromatic beam of 30 keV, cropped to obtain a pencil-beam of 50 x 50 μm. The images were recorded using a FReLoN 2k camera with a binning factor of 2, and an optic system producing images with an isotropic pixel size of 40 μm. The thin section was positioned perpendicular to the beam, and we analysed a 2.2 x 2.2 mm portion (S2 Fig). This portion was characterized step-by-step, recording independent diffraction pattern at individual points of the whole portion (89 x 89 points). Each diffraction pattern was obtained from 2 seconds of exposure and the thin section was displaced by 25 μm, either horizontally or vertically, to create a diffraction mapping. It resulted in 7921 diffraction patterns. Because for each recorded image, only a few crystals were oriented in Bragg conditions, we produced maximum intensity projections of several diffraction images representing various area of the studied portion. From these projection images, we performed 360° azimuthal integration, resulting in a plot of intensity of the diffractions circles (represented as peaks) versus distance to the centre of the image, in pixel. As the wavelength of the incoming X-ray beam was quite unusual (0.413 Å compared to more common 1.54 Å obtain from Cu Kα) it was decided to convert the distance to the centre values into d-spacing values rather than 2θ angles. The resulting diffraction patterns were compared with standards from the literature to identify dominant mineral phases.

## Results

### Results from the scans and virtual extraction of the embryonic material

**Fig 7:**
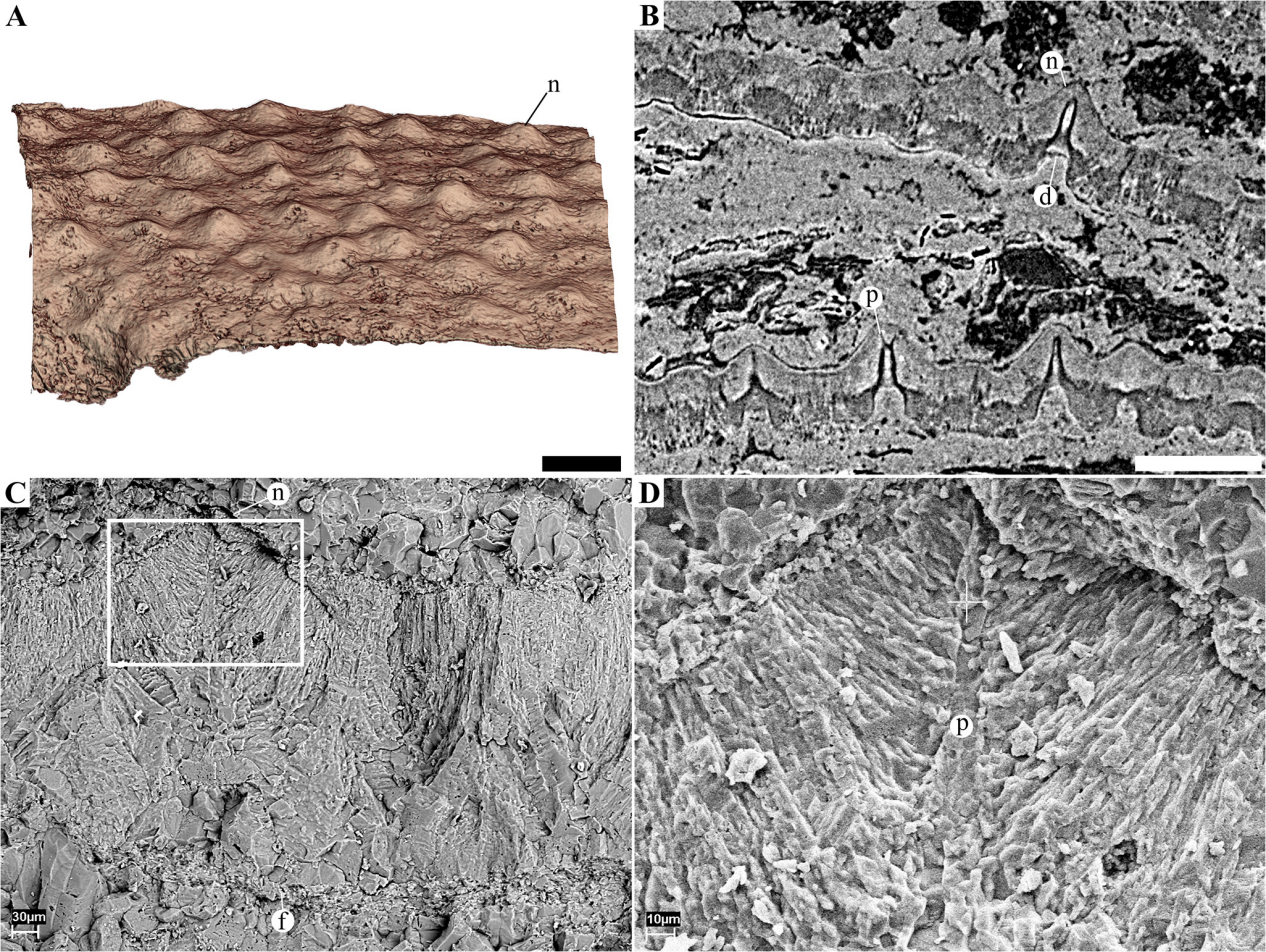
Eggshell morphology and microstructure of the eggs from Phu Phok. **A**, 3D rendering of a portion of the surface of the eggshell of SK1-2 showing the distribution of nodes. **B**, tomogram of SK1-1 showing two eggshell fragments that slid in the egg, outer surfaces oriented to the top of the figure. The inner half of both shell fragments is displayed in darker shades of grey indicating the shell is less dense than the whiter outer half. Unlike **micrographed thin sections (Fig** 7), the funnel-shaped depression (d) do not seem to be obstructed. The pore canals (p) are highlighted by the edge interference resulting from the phase contrast effect (black and white fringes). **C-D**, SEM photographs of an eggshell fragment showing the fan-shaped pattern of crystal at the level of a surface node (n). Not the fibrous layer (f) underlining the eggshell. **D**, close up from C. Scale bars (A, B), 500 μm.

Together, the embryonic remains contained in the eggs SK1-1 and SK1-2 represent almost the full skeleton. Both of these specimens exhibit an exceptional level of bones preservation, but almost no anatomical connection of the skeleton (Fig 2). From the specimen SK1-1, we extracted 378 skeletal elements (including 77 unidentifiable fragments, Fig 2A and S1A–S1C Fig). The remains are composed of 17 bones from the skull, 2 calcified endolymphs from the sacculus (Fig 5B) and 7 mandibular bones; 72 presacral (23 centra and 49 neural arch elements) and 66 caudal (34 centra elements and 32 neural arch elements) vertebrae; 63 ribs (complete or partial); 8 pectoral bones; 66 limb bones (including 2 humeri, 6 zeugopodia; and 48 autopodial bones). The mandibles show exceptional preservation but are incomplete due to the erosion of the corresponding part of the egg. The skull is mostly represented by all neurocranial bones including a fragmentary parietal, with the exception of the missing opisthotic. The other preserved skull elements include the pterygoids and a quadrate. The pectoral girdle was found to be nearly complete (Fig 5H), and so are the forelimbs (at least the humeri, a radius and an ulna). However, it has been difficult to discriminate between manual and pedal phalanges (Fig 6). The pelvic girdle and hindlimbs are absent.

From the specimen SK1-2, we extracted 583 skeletal elements (including 220 unidentifiable fragments, Fig 2B and S1D–S1F Fig). The diagnosable remains are composed of 56 bones from the skull, 2 calcified endolymphs from the sacculus, 14 mandibular bones and 7 isolated teeth; 114 vertebral elements (61 centra and 53 neural arch elements); 65 ribs (complete or partial); 9 pectoral bones; 6 pelvic bones; 72 limb bones (including 4 stylopodia, 7 zeugopodia and 61 autopodial bones). This specimen is more complete than the later but its bones are in general more fractured. The skull is nearly complete including facial, palatal and neurocranial bones (Figs 3 and 4). The craniofacial bones are better preserved on the left side. The palatal region is complete. The neurocranium is poorly preserved. All mandibular bones are present and almost complete, and the dentary bears about 17 teeth (17 teeth are preserved in association with the right dentary; only 13 have been discovered associated to the left one). The overall position of the skull bones inside the egg is roughly reminiscent of the original anatomical position (however, the right dentary is turned at 180 degrees relative to the general position of the bones). The pectoral girdle consists of a set of poorly ossified bones (Fig 6). The forelimbs are represented by the presence of the humeri, ulna and radius. As for the SK1-1 egg, it has been difficult to discriminate between manual and pedal phalanges (Fig 6). The pelvic girdle is complete except the ischii that are crushed proximally (Fig 5I). The hindlimbs are represented by a femur and paired tibia and fibula

The two eggs SK1-3 and SK1-4 are considerably crushed but both of them contain embryonic remains [16]. Based on the microtomographic data, the preservation of the embryos seems to be of a lower quality than in SK1-1 and SK1-2. Because of their preservation, it was decided not to segment them.

The examination of the data produced from the scan of SK1-6 revealed the presence of an embryo with a very bad preservation pattern, displaying hundreds of mixed bone fragments. As it appeared that it would not be really helpful for the present study, it was decided not to segment it. Finally, the scan of SK1-7 showed no embryonic remains preserved inside this egg.

### Osteological description

The reconstruction of the cranium based on material from both eggs portrays a long and slender skull ending in a pointed snout (Figs 3 and 4). The unsculptured dermatocranium is characterized by retracted nares, narrow paired frontals strongly widening caudally and poorly ossified parietal (Fig 3A). Being only preserved in the oldest embryo (SK 1–1), it is unclear if the parietal consists of a single bone or two paired elements fusing by their medial caudal margin. The orbit is dorsally bounded by the subolfactory process projecting as a simple ventral downgrowth (Figs 3B and 4B). Ventrally, the orbit is exclusively bordered by an elongate and curved jugal which partly overlaps the caudal process of the maxilla and contacts the prefrontal rostrally (Fig 3B). The broad and high maxilla includes a row of 12 broadly spaced teeth located anterior to the orbit (Fig 4C). The dorsal process of the maxilla overlaps the rostral facet of the prefrontal, contacting the rostral part of the frontal. The prefrontal marks the rostral margin of the orbit with a broad orbitonasal flange. A ventrolateral process of the prefrontal contacts the rostral tip of the jugal. The palate is characterized by a wide interpterygoid vacuity which is bordered by edentulous bones (Fig 4A). The palatine, as long as wide, presents a broad and elaborated contact with the prefrontal and slightly connects caudally the long and slender pterygoid (Fig 4B). The rudimentary epipterygoid from SK1-2 consists of a simple shaft (Fig 3B). In the splanchnocranium, the well ossified quadrate displays a highly developed tympanic crest but lacks the cartilaginous dorsal and ventral condyles (Fig 3B). Remarkably, elements of the sclerotic ring are preserved in SK1-2, some of them in anatomical connection (Fig 5A) and also the ossified part of the columella-apparatus is partly preserved in SK1-1 (Fig 3B).

The reconstruction of the embryonic neurocranium displays a relatively large braincase with rudimentary processes (S3 Fig). Due to the early ontogenetic stages of both embryos, joints between bones of the neurocranium are separated by wide areas, which represent the unpreserved growing cartilage. In the dorsal part of the braincase, the supraoccipital is rather rostrocaudally short due to the elementary development of the tectum synoticum and does not display a clear dorsal sagittal crest (Fig 3A). Remarkably, the calcified extracranial endolymph sacs of both specimens are extensively developed, extending outside the dorsal limit of the parietal (Figs 3A, 3B and 4B). In the rostral region of the braincase, the prootic displays an incipient alar process and a large trigeminal notch (Fig 4B). The incipient crista prootica poorly overlies the well marked recessus vena jugularis which contacts a single external facial foramen visible in both lateral and medial views (S3 Fig). The ventral part of the braincase is delimited by a rather flat parabasisphenoid (Fig 4A). Caudolateral flanges of the latter overly the basioccipital. The incomplete suture between these two bones is marked by the presence of a basicranial fontanelle. The rostral part of the parabasisphenoid is characterized by short basipterygoid processes and laterally placed anterior opening of the Vidian canal located in the floor of the braincase, slightly dorsal to the parasphenoid rostrum (S3 Fig). The posterior opening of this canal is ventrally bordered by the parabasisphenoid and dorsally by the prootic. In the caudal region of the neurocranium, the opisthotic and exoccipital are unfused (Fig 4A). The paraoccipital process of the opisthotic being broken, it is unclear how this structure was extending.

The reconstruction of the mandible was obtained from the younger embryo (SK1-2) as this egg preserved all mandibular bones (Figs 3C and 4C). The mandible is low, slender and straight and bears numerous widely spaced teeth (17 counted for SK1-2, Fig 4C). The rostral part of the mandible displays a shallow rostrally open and ventrally oriented Meckelian canal (Fig 4C). The splenial encloses this canal as anterior as to the 2/3 of the dentary, and ends caudally before the coronoid apex (Fig 4C). The splenial is solely involved in the formation of the anterior alveolar foramen. Laterally, the dentary overlaps broadly the post-dentary unit and displays a clear notch separating the surangular and angular processes (Fig 3C). Medially, the contact is loose as the dentary only slightly overlaps the rostral ramus of the coronoid. The caudal portion of the mandible is ventrally bordered by the coossified articular and prearticular (Fig 4C). The surangular is rostrodorsally expanded and presents a distinct surangular foramen.

Teeth are only preserved in the younger embryo (SK1-2) which exhibits a pleurodont dentition. There are two morphotypes of teeth: those in more rostral positions are conical with an expanded base (Fig 5C). They are trenchant (two incipient carinae on the medial and the lateral sides were observed, Fig 5C), some of them being slightly recurved. The second types of teeth are located more caudally. They are wider rostrocaully and more flattened mediolaterally. The base of the teeth is quite constant in diameter and tapers suddenly close to the tip.

In both specimens, the postcranial skeleton is remarkably less ossified than the cranium but several bones exhibit interesting features (Figs 5 and 6). A precondylar constriction of the centrum is observed on presacral vertebrae (Fig 5D). The neural arches exhibit low and poorly developed zygapophyses. In SK1-2, several neural arch elements bear a well developed transverse process. The caudal vertebrae have an autotomous plane caudal to the transverse process (Fig 5E and 5G). In the pectoral girdle, the clavicles show a remarkable expansion (Fig 5H). In the pelvic girdle, the ilia show a rather short and narrow blade and a small cranial tuberosity (Fig 5I). Most of the limb bones consist of simple shafts lacking ossification of the extremities preventing a relevant description (Fig 6). Remarkably, the ungula phalanges are well developed and claw shaped with a flat ventral surface.

### Eggshell description

**Fig 8:**
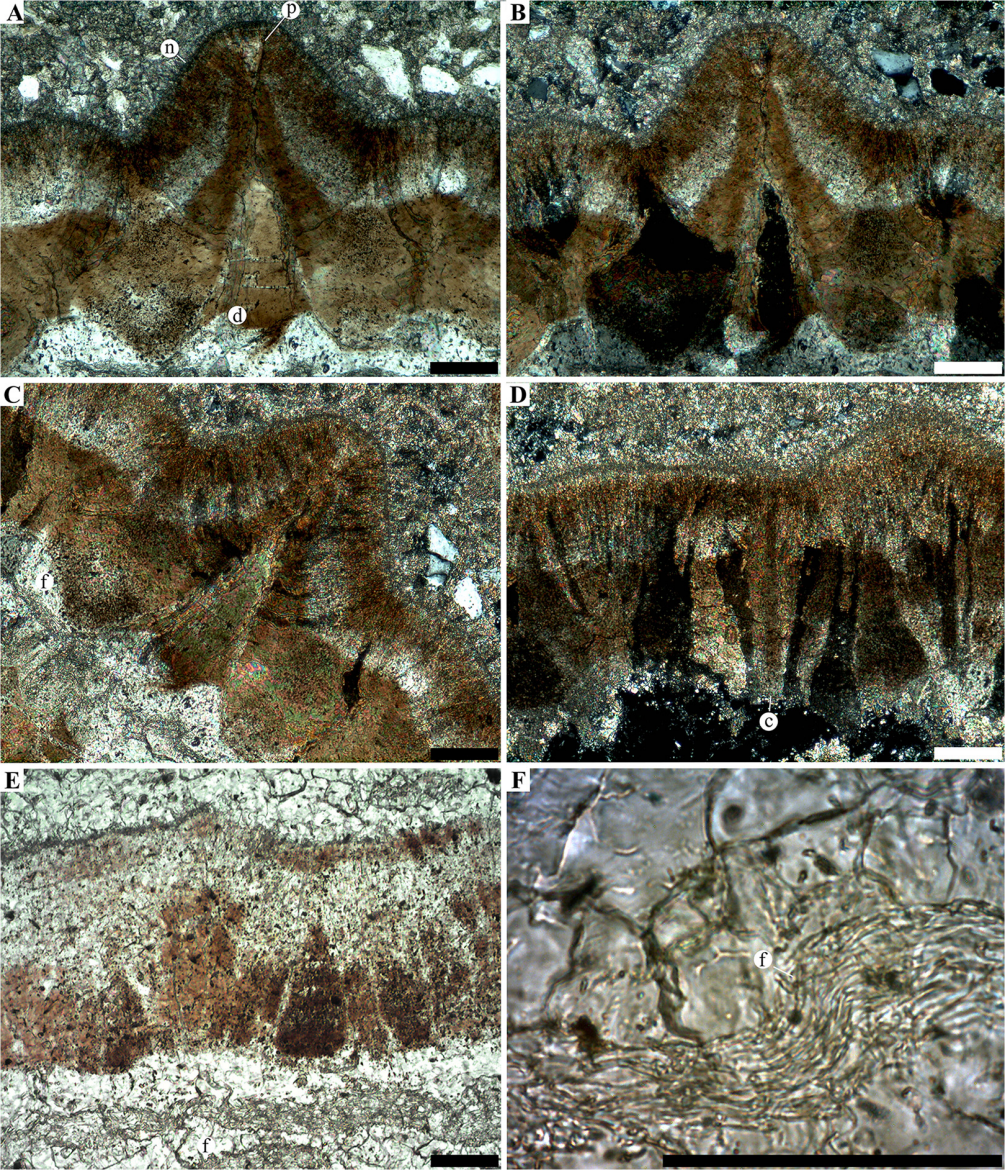
Micrographed radial thin section of egg SK1-5. **A-C**, close up on a radial section at the level of a tall ornamentation node (n) in non-analysed polarized light (A) and analysed polarized light (B and C). The funnel shaped depression (d) exhibit similar interference colours than the innermost part of the shell. The depression tapers toward the outer surface into a very narrow pore canal (p). **D**, flat portion of the eggshell in analysed polarized light showing large crystals (c) with a columnar extinction. **E**, flat portion of the eggshell in transmitted light, showing the eggshell underlined by a fibrous layer (f). **F**, close up on the fibrous layer. The outer surface of the eggshell is positioned on the top part of each panels (top-right in panel C). Scale bars, 100μm.

All discovered eggs are markedly crushed, with only SK1-1 being preserved well enough to assess the original size and shape (Fig 1), as was already noted on a previous study [11]: the eggs were most likely ellipsoid in shape, measuring 18 mm in height and 11 mm for maximum diameter, giving an estimated volume of 1.15 cm^3^. The outer surface of the shell is marked by a bimodal nodular ornamentation (Figs 1 and 7A), with the taller nodes being more common than shorter ones. Opposite to the nodes, the eggshell is pierced by funnel-shaped depressions (Figs 7 and 8). The depression tapers into a narrow canal which exits through the tip of superficial node. We interpret these structures as non-branching pore canals. No concretions have been observed attached or in close association with the inner depressions. These depressions are best visible on microtomographic data (Fig 7B) while they seem obstructed on micrographed thin sections (Fig 8) and SEM (Fig 7C). On micrographed thin section, the obstructing material shows an interference colour similar to these of the eggshell and difference of the surrounding sedimentary matrix (Fig 8). It suggests the inner depressions were filled by precipitation, remobilizing material of the eggshell. Unlike previous descriptions [11,21], we did not observe divisions of the eggshell into distinct crystalline layers. From thin section, SEM and microtomographic data, the eggshell consists of a continuous single crystalline layer generally underlined by another layer fibrous in aspect (Fig 8E and 8F).

**Fig 9:**
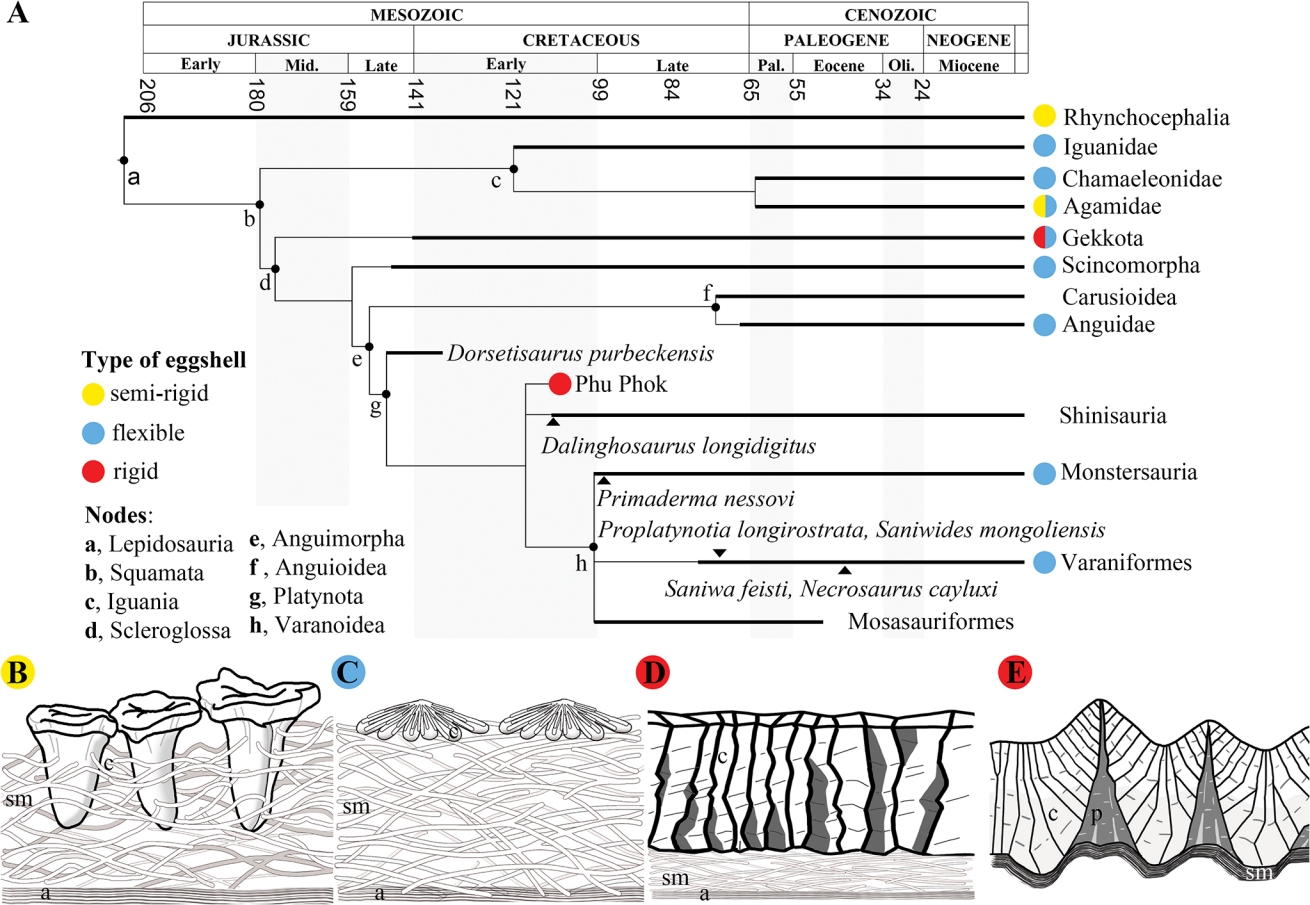
Known eggshell types across a simplified time-calibrated lepidosaur phylogeny based after morphological studies [28,31]. **A**, Phu Phok embryos are tentatively placed in an unresolved trichotomy with shinisaurids and varanoids. **B-E**, schemas of known lepidosaur eggshell types: semi-rigid, loosely connected calcite columns embedded in the shell membrane [36,47] (B, modified from Packard et al. [35]). Examples: Tuatara (Rhynchocephalia: *Sphenodon punctatus*)[35,36] and Bearded lizard (Agamidae: *Pogona barbata*)[47]; leathery, shell membrane often covered with thin calcitic elements [8]. Examples: the wall lizard (Scincomorpha: *Lacerta lepida*)[9], zebra-tailed lizard (Iguania: *Callisaurus draconoides*)[38]; (C); rigid, well-connected adjacent calcitic columns covering a thin shell membrane [6,7,9]. Example: *Gekko gecko*[7]; (D); Phu Phok, similar to the rigid type, developed in an undulatory pattern, covering a thin structure interpreted as the shell membrane (E). Abbreviations: a, amorphous layer; c, calcite component; p, pore canal; sm, shell membrane.

The X-ray diffraction pattern from the eggshell indicates it is composed of a compact matrix of large crystals of calcium carbonate in the form of calcite (S2 Fig). The large crystals are particularly visible from micrographs of thin sections under cross-polarized light, displaying a columnar extinction pattern (Fig 8). On the flat portion of the shell (i.e., between ornamentation nodes), the columnar crystals are approximately vertical (Fig 8D). At the level of nodes, the calcitic columns are organized in a fan-shaped pattern, converging toward the central pore canal (Figs 7C–7D and 8A–8C). When looking at two contiguous nodes in thin section, the crystalline pattern is not clearly defined between the two neighbouring fan-shaped structures. These in-between zones extinct as a whole under cross-polarized light (Fig 8B). In general, the calcitic columns display a undulating pattern, almost vertical on flat portion of the shell, and in a fan shape at the level of nodes (Figs 7–9).

From the micrographed thin sections in non-analyzed polarized light, the lower half and the upper quarter are dark with a brown colour. By contrast, the remaining interval displays a light tone. The lower dark portion is also displayed in dark grey level on the micrographic data while the whole upper portion is characterized by lighters shades, denoting a clear difference in X-ray attenuation (Fig 7B). The limit between the two portions is also underlined by a dark and white fringe resulting from the phase shift effect. Both the grey level contrast and the edged enhancement indicate a clear density variation. From the X-ray diffraction analyses, these parts displayed in different colours did not show variation of crystal phase, but solely calcite.

Finally, another zone can be considered: underlying the calcareous shell, a thin structure follows the inner surface (Fig 8E and 8F). This structure has a fibrous aspect, especially visible on highly magnified micrographed thin sections, and is either directly connected to the calcareous part or slightly detached. The fibrous aspect and the position (i.e., underlying the calcareous component of the eggshell) strongly suggest it represents the shell membrane, an organic shell present in squamate eggs. We did not notice cores of calcification either at the contact with the shell membrane and the calcitic shell or in the shell membrane.

From the microtomographic data we measured the shell thickness without the ornamentation, the prominence of both types of nodes and the thickness of the fibrous structure underlying the eggshell. For SK1-1 the shell thickness averages 331 μm (number of measurements (N) = 13; Standard Deviation (SD) = 15 μm; Range = 306–360 μm; Median (M) = 333 μm); tall nodes average 122 μm (N = 11; SD = 9μm; range = 108–138 μm; M = 121μm); short nodes averages 87 μm (N = 4; SD = 4μm; range 78–86 μm; M = 85μm); and the fibrous layer averages 40 μm (N = 5; SD = 5 μm; range = 33–46 μm; M = 40 μm). For SK1-2 the shell thickness averages 286 μm (N = 10; SD = 11μm; range 264–299 μm; M = 287μm); tall nodes average 185 μm (N = 16; SD = 12μm; range 162–202 μm; M = 188 μm); short nodes average 108 μm (N = 5; SD 16 μm; range 92–128 μm; M = 103 μm).

Shell units are not obvious in cross section and no structure permits to distinguish such structures from examination of the external or the internal surface. The fan-shaped arrangement at the level of the nodes could be interpreted as single shell unit. However, as the calcitic columns do not radiate from a single point it is unlikely that it represents individual shell units. No accretion lines are visible within the columnar part of the shell.

## Discussion

The digital segmentation of the two least crushed eggs (SK1-1 and SK1-2) [16] shows that both embryonic skeletons are mostly disarticulated, but assembled into clusters reflecting the original position (Fig 2 and S1 Fig). The embryonic remains from the egg SK1-1 lack the rostral part of the skull, the pelvic girdle and hind limbs but include well preserved elements notably from the braincase and the pectoral girdle (Figs 3–5); SK1-2 lacks the rostral right part of the skull but preserves most craniofacial bones, complete mandibles with teeth, the pelvic girdle, and hind limb bones (Figs 3–5). Both embryos represent advanced but distinct developmental stages as reflected by their different degrees of ossification (Fig 6): The oldest (from egg SK1-1) displays an advanced level of ossification notably marked by the onset of fusion of the basi- and exoccipitals (Fig 4A), sutures between all postdentary bones and fully ossified retroarticular processes. These features suggest that the animal was likely close to hatching [22]. The youngest embryo (from SK1-2), shows well developed articulations of the frontal with pre- and postfrontals (Fig 3A), and of the palatine with the maxilla and the prefrontal (Fig 4A and 4B). Limb bones of both specimens are poorly ossified, consisting of simple shafts lacking epiphysial caps (Fig 6).

Among anatomical characters relevant for such early ontogenetic stages [23], the two embryos from Phu Phok display numerous features denoting anguimorph affinities (See S1 Table for a summarized comparison of the Phu Phok embryos with several anguimorph taxa): Meckelian canal opening rostral to the splenial (Fig 4C); long horizontally oriented rostral extension of the coronoid (Fig 4C); absence of a tubercle on the medial surface of the retroarticular process [24–26] (Fig 4C); and pleurodont teeth borne by a single, inclined surface [27] (Fig 4C). This identification is further supported by additional synapomorphies such as ossified chevron-shaped palpebrals [25]; Meckelian canal subdivided by intramandibular septum near the caudal end of the dentary tooth row [25] (S3 Fig); developing teeth single-cusped [28] (Fig 4C), some with a somewhat recurved crown [24]; and no more than 13 teeth in the maxilla [26] (Fig 4C). Among anguimorphs, the embryos show three autapomorphic characters such as the anterior inferior alveolar foramen solely formed by the splenial (Fig 4C), a broad palatine-groove articulation of the prefrontal (Fig 3A) and autotomy planes present posterior to the transverse process (Fig 5G). These unique characters combined with their geographical and stratigraphical provenance [29,30] suggest that the embryos belong to a hitherto unknown lizard. The only additional squamate material from the Sao Khua Formation consists of two vertebrae from the same locality (Fig 5F). The latter specimens are positively assigned to Anguimorpha on the basis of long, narrow centra with concave lateral margins and clearly posterodorsally facing condyles. Whether these vertebrae represent the adult counterpart of the embryos is not ascertainable without further adult remains. Despite an advance state of ossification of these embryos, the description of additional characters of taxonomical importance would not be reliable at these ontogenetic stages. Together with the absence of an unambiguous association with adult material, it prevents a definitive and precise diagnosis and therefore, the erection of a new taxon.

Within the traditional view of the Anguimorpha clade, i.e. Anguioidea and Platynota, sensu Conrad [28] (see Fig 9), the embryos display a variety of features pointing notably to Platynota [28]. For instance, the maxillary tooth row located anterior to the orbit (Fig 3B) is present in most platynotans but shinisaurids and the basal *Dorsetisaurus* [24,31]. The absence of a subdental shelf on the dentary (Fig 4C) is characteristic of most platynotans with the exception of a few Mosasauriformes and *Dorsetisaurus* [31]. The long and low rostral process of the coronoid (Fig 4C) is a synapomorphy of varanoids [31]; the caudal margin of the splenial ending at (or rostral to) the coronoid apex (Fig 4C) prevails among platynotans, omitting the shinisaurid *Dalinghosaurus* [25,31]. Widely spaced acute teeth with expanded bases (Fig 4B and 4C) characterize platynotans but are absent in *Dorsetisaurus* and shinisaurids [24,31]. The presence of a cranial process on the ilium (Fig 5I) is a synapomorphy of varanines also observed in *Dalinghosaurus* and the varaniform *Telmasaurus* [31].

Despite the presence of features suggesting affinities with derived platynotans (Fig 9, S1 Table), the Phu Phok embryos also present characters conflicting with this clade. The caudoventral process of the jugal (Fig 3B), predominant in Anguioidea, Monstersauria and Shinisauridae, is absent in Varaniformes except ‘*Saniwa’ feisti* and most Mosasauriformes [28,31]. The plate-like vomer (Figs 3A and 4A) differs from the rather rod-like shape present in platynotans except *Shinisaurus*, *Lanthanothus* and *Proplatynotia* [31]. In the braincase, caudolateral flanges of the parabasisphenoid overlap the basioccipital (Fig 4A), an anguimorph synapomorphy which tends to disappear in Varaniformes (*Lanthanotus* and *Varanus*) [31,32]. The caudolateral margin of the dentary is forked into the coronoid and surangular processes (Fig 3C), as in most Anguioidea, several basal platynotans including *Dorsetisaurus*, *Shinisaurus* and *Dalinghosaurus*, and the varaniform *Proplatynotia* [24,31]. The cranial caudal vertebrae of the Phu Phok embryos retain autotomic planes permitting self-amputation of the tail (autotomy, Fig 5E and 5G). Although present in most closely related anguids, this character is absent in platynotans with the exception of all shinisaurids, the monstersaurid *Primaderma* and the mosasaurid *Dolichosaurus* [28,31]. In the pectoral girdle (Fig 5H), the cranial process of the interclavicle is also present in most anguids and two shinisaurids (*Shinisaurus* and *Dalinghosaurus*) and absent in *Varanus* [31].

The embryos from Phu Phok display an interesting mixture of characters within Anguimorpha notably by the co-occurrence of shinisaurid, helodermatid, varaniform and mosasauriform synapomorphies, sensu Conrad [26] (Fig 9). While adult material would be necessary for extensive comparisons, gross anatomical observations firstly denote closer affinities with these taxa than with Anguioidea [31]. Consequently, they suggest that these embryos represent at least a stem group of Platynota, sensu Conrad [26], strengthening the phylogenetic validity of this clade, unlike recent phenotypic cladistic analyses [31,33].

The taxonomic conclusion of the Phu Phok embryos as squamate conflicts with the previous interpretation on these specimens as theropod based on eggshell analyses [11]. New data on the microstructure of the eggshell (Figs 7 and 8) show numerous characters comparable with rigid-shelled eggs of modern Gekkonids [6,7,9]: a single thick (~350 μm) calcareous and continuous layer consisting of interlocking columns forming a compact crystalline matrix; outer surface dotted with nodes; inner surface with several funnel-shaped depressions; narrow channels connecting the inner depressions to the outer nodes. As observed in modern gekkonid eggs, these narrow channels constitute the pore system used for gas exchanges designed to maintain a low conductance compared to other rigid-shelled eggs (e.g., birds, crocodiles) [6–8]. The inner depressions on modern gekkonid eggs are caused by the detachment of large calcitic concretions [6,7], which is unlikely for the Phu Phok eggs as, 1) no isolated concretions were observed in these eggs; and 2) the arrangement of the columns follows the undulation of the shell and are almost perpendicular to the pore channels (Fig 8). These observations suggest that the formation of outer tubercles is somehow linked to the development of the inner depressions, resulting in an undulating pattern unique among lepidosaurs.

## Conclusions

The discovery of anguimorph embryos inside rigid-shelled eggs was rather unexpected as this mode of reproduction was thought to be an exclusive specialisation of gekkonid among squamates [5,7–9,34] (Fig 9). The similarities observed between the rigid-shelled eggs of modern gekkonids and the Phu Phok anguimorphs are likely the result of an evolutionary convergence as leathery-shelled eggs are predominant in all other squamate clades [5,35,36] (Fig 9). Unlike rigid-shelled eggs, eggs of most oviparous squamates present a leathery aspect which consists of a variable and thin coating of calcite overlying a fibrous shell membrane [35]. The rigid type of eggshell presents a similar pattern but differs in having a thicker calcitic layer, allowing notably oviposition in drier environments [37]. The squamate oviduct is known to produce eggs with variable amount of calcite, even at the intraspecific level [38]. This modularity in calcite secretion is considered as one of the key aspects that lead to egg retention through thinning of the calcitic layer, in most major squamate clades [34]. The Phu Phok anguimorphs, on the other hand, demonstrate that the plasticity of the oviduct bears the possibility to increase the calcitic component which occurred at least twice over the evolution of squamates. Consequently, while rigid-shelled eggs produced by squamates present a unique microstructure among amniotes [34], it is currently impossible to retrieve more detailed taxonomical information from fossilised isolated eggshell of squamates.

Taxonomical interpretation of isolated eggs based on eggshell microstructure has lead to misidentification on several occasions [39–41]. More recently, a new approach based on egg geometry also concluded that taxonomical identification of fossil eggs based on the shape could be problematic [42]. While the presence of embryonic remains seems the less questionable way to address a taxonomical identification, poorly ossified embryonic material can lead to a limited taxonomical identification or misinterpretation [43–46]. Eventually, only exceptional preservation of well-ossified embryonic material provides adequate taxonomical information to address questions on the evolution of squamate reproduction modes.

## Supporting Information

S1 Fig Three-dimensional rendering of the embryonic remains contained in the egg SK1-1 (A-C) and SK1-2 (D-F) rotated at 90 (A, D), 180 (B, E) and 270 (C, F) degrees relative to orientation in Fig 2. Colour codes: red, skull and mandible; yellow, vertebrae; grey, ribs; green, pectoral and pelvic girdle; blue, limbs. Scale bar, 1 mm.
(TIF)

S2 Fig X-ray radiography and X-ray diffraction data from SK1-5. **A**, digital image of the thin section of SK1-5 in transmitted light overlaid by stitched radiographs to which a flat-field correction and a single distance phase retrieval filter (ANKA phase [17]) have been applied. **B**, plot of inverted log d-spacing versus square root of intensity (normalized by maximal value of from each set of data) showing diffraction pattern of the whole scanned portion (total) and 3 regions of interest: reg, eggshell zone 1; green, eggshell zone 2; blue, matrix zone 3. Mains diffraction peaks of calcite [48] and quartz [49] are indicated on the bottom part as well as associated Miller indices (C—hkl, calcite; Q—hkl, Quartz), marked on peaks of the total diffraction pattern. Several peaks remained unidentified, suggesting the presence of at least another mineral phase. Scale bar, 1 cm. [48,49].
(TIF)

S3 Fig Interactive 3D rendering of the reconstructed skull and mandibles based on bones from both specimen SK1-1 and SK1-2. Default view displays the skull and mandibles in an antero-latero-dorsal view, with a perspective projection mode. Bones are grouped in four categories (craniofacial, neurocranium, viscerocranium, mandibles).
(PDF)

S1 Table List of character states discussed to interpret the phylogenetic affinities of the embryos from Phu Phok compared with various anguimorph taxa. Columns represent states of character for the Phu Phok embryos and taxa amongst anguimorph. For taxa preceded by the mention ‘mode’, the state of character utilised is the mode (i.e., most common value) from the matrix of Conrad et al. [31]. When two states of character were equally distributed, both states are represented inside brackets. Rows describe the different states for the considered characters (numbers inside bracket indicate the character number utilised by Conrad et al. [31]). Characters are selected and sorted to highlight phylogenetic affinities of the embryos from Phu Phok. Abbreviations: A., Anguidae; Angui., Anguioidea; C., Carusioidea; M., Mosasauriformes; Monst. Monstersauria; Shini., Shinisauria.
(DOCX)
