# Supplementary figures and images for: Evidence of Egg Diversity in Squamate Evolution from Cretaceous Anguimorph Embryos
Source: PLoS One. 2015 Jul 15;10(7):e0128610. doi: 10.1371/journal.pone.0128610 (PMC4503689; doi:10.1371/journal.pone.0128610)

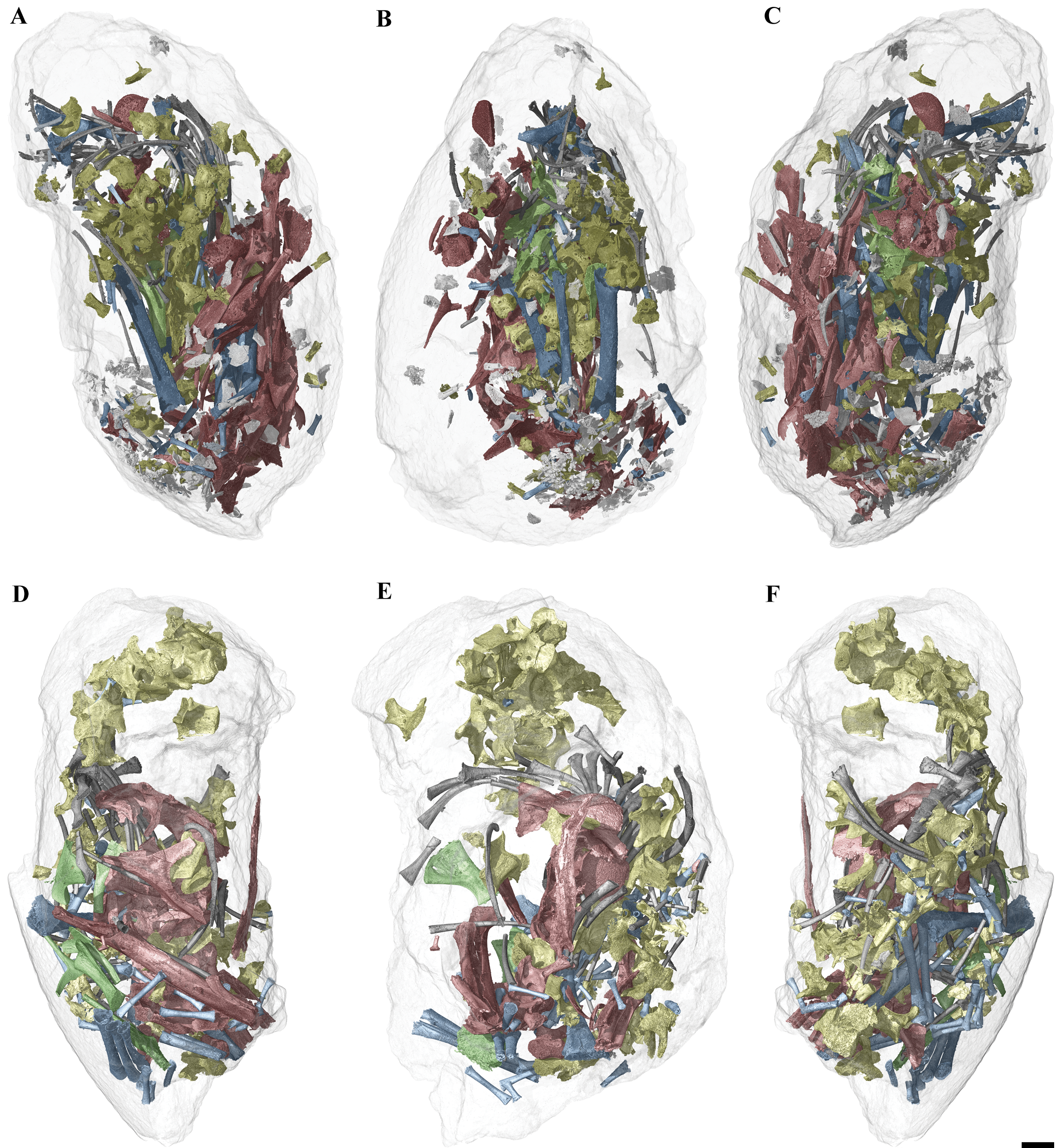

Supplement: S1 Fig — Colour codes: red, skull and mandible; yellow, vertebrae; grey, ribs; green, pectoral and pelvic girdle; blue, limbs. Scale bar, 1 mm. (TIF) [file pone.0128610.s001.tif]

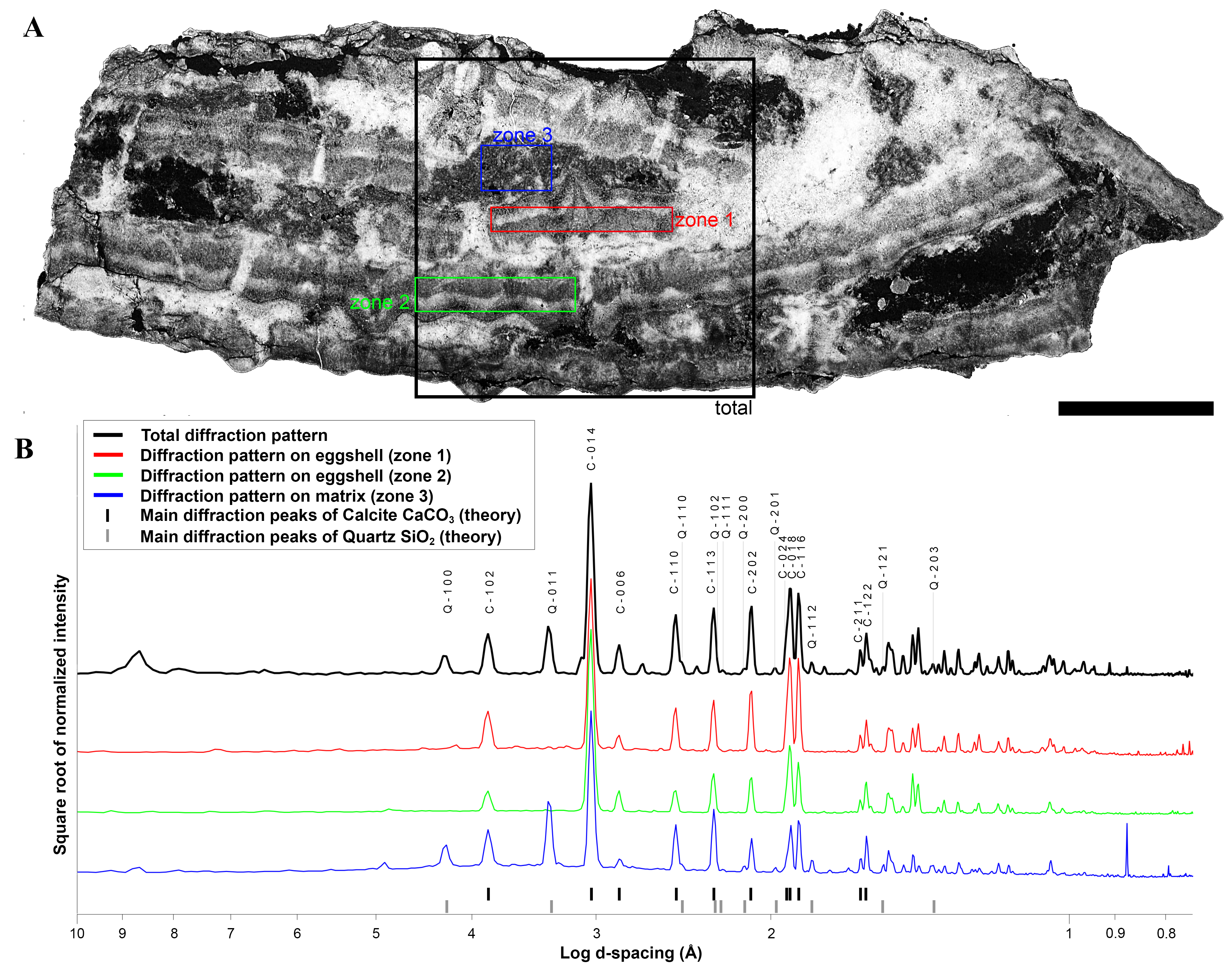

Supplement: S2 Fig — A, digital image of the thin section of SK1-5 in transmitted light overlaid by stitched radiographs to which a flat-field correction and a single distance phase retrieval filter (ANKA phase [17]) have been applied. B, plot of inverted log d-spacing versus square root of intensity (normalized by maximal value of from each set of data) showing diffraction pattern of the whole scanned portion (total) and 3 regions of interest: reg, eggshell zone 1; green, eggshell zone 2; blue, matrix zone 3. Mains diffraction peaks of calcite [48] and quartz [49] are indicated on the bottom part as well as associated Miller indices (C—hkl, calcite; Q—hkl, Quartz), marked on peaks of the total diffraction pattern. Several peaks remained unidentified, suggesting the presence of at least another mineral phase. Scale bar, 1 cm. [48,49]. (TIF) [file pone.0128610.s002.tif]

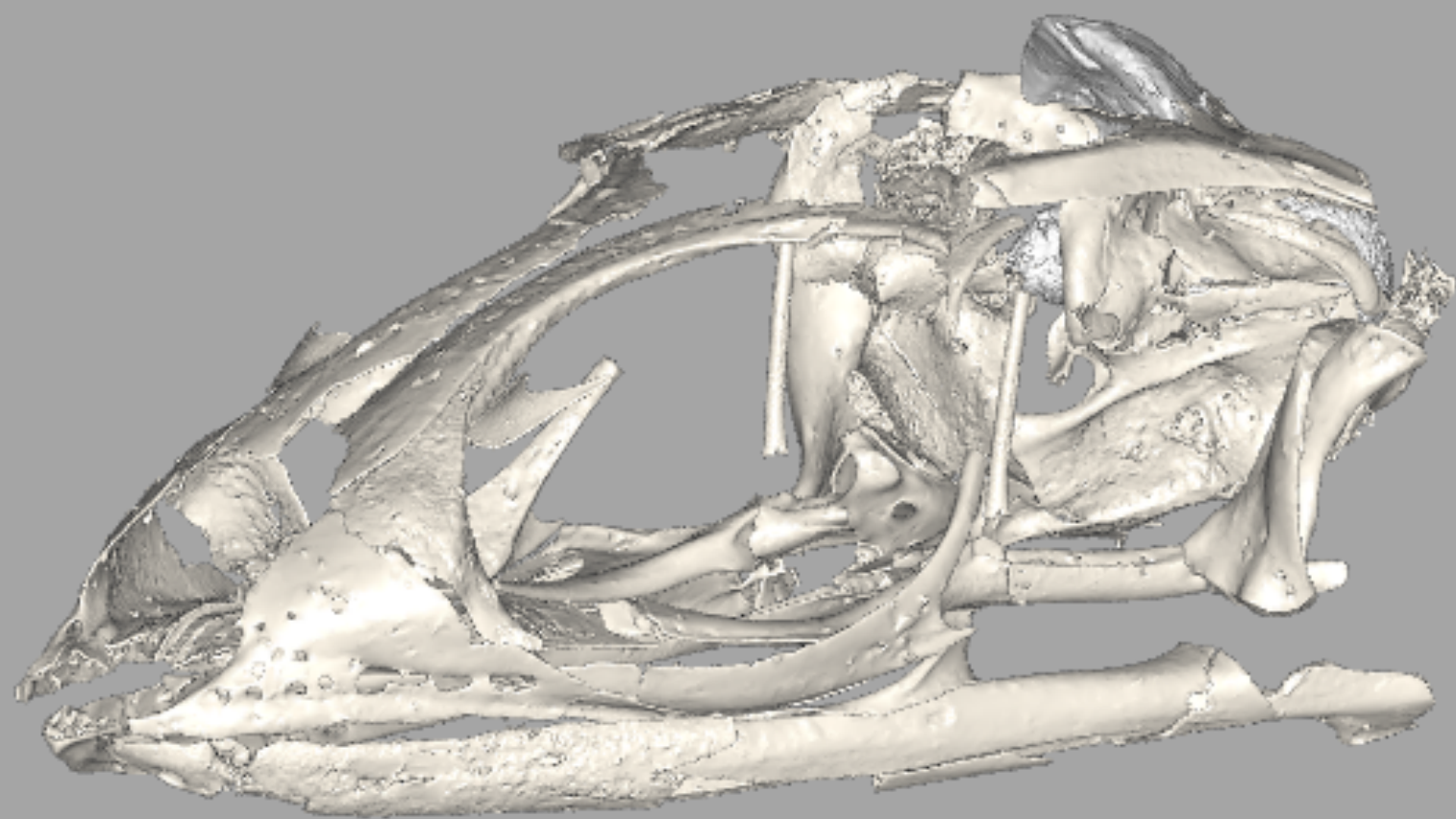

Supplement: S3 Fig — Default view displays the skull and mandibles in an antero-latero-dorsal view, with a perspective projection mode. Bones are grouped in four categories (craniofacial, neurocranium, viscerocranium, mandibles). (PDF) [file pone.0128610.s003.pdf]
