# Supplementary material for: Evidence of Egg Diversity in Squamate Evolution from Cretaceous Anguimorph Embryos
Source: PLoS One. 2015 Jul 15;10(7):e0128610. doi: 10.1371/journal.pone.0128610 (PMC4503689; doi:10.1371/journal.pone.0128610)
Supplement: S1 Table — Columns represent states of character for the Phu Phok embryos and taxa amongst anguimorph. For taxa preceded by the mention ‘mode’, the state of character utilised is the mode (i.e., most common value) from the matrix of Conrad et al. [31]. When two states of character were equally distributed, both states are represented inside brackets. Rows describe the different states for the considered characters (numbers inside bracket indicate the character number utilised by Conrad et al. [31]). Characters are selected and sorted to highlight phylogenetic affinities of the embryos from Phu Phok. Abbreviations: A., Anguidae; Angui., Anguioidea; C., Carusioidea; M., Mosasauriformes; Monst. Monstersauria; Shini., Shinisauria. (DOCX) [file pone.0128610.s004.docx]

**Table 1.** List of character states discussed to interpret the phylogenetic affinities of the embryos from Phu Phok.

|  |  |  | **Angui.** | | **Platynota** | | | | | | | | | | | | |
| --- | --- | --- | --- | --- | --- | --- | --- | --- | --- | --- | --- | --- | --- | --- | --- | --- | --- |
|  |  |  | **C.** | **A.** |  | **Shini.** | | **Monst.** | | **Varaniformes** | | | | | | | **M.** |
|  |  | **Phu Phok embryos** | **Mode: Carusioidea** | **Mode: Anguidae** | *Dorsetisaurus purbeckensis* | **Mode: Shinisauria** | *Dalinghosaurus longiditus* | **Mode: Monstersauria** | *Primaderma nessovi* | **Mode: Varaniformes** | **Mode: Lanthanotinae** | **Mode: Varaninae** | *Necrosaurus cayluxi* | *Proplatynotia longirostrata* | *Saniwides mongoliensis* | *Saniwa feisti* | **Mode: Mosasauriformes** |
| Anguimorpha affinities | Palatine, teeth (0: pre, 1: abs, 2: single line) [115] | **1** | 1 | 1 | 0 | {01} | 0 | 1 |  | 1 | 1 | 1 | 2 | 1 | 0 |  | 1 |
|  | Parabasisphenoid, Ant. opening of Vidian canal (0: ventral to dorsum sellae) [145] | **0** | 0 | 0 |  | 0 | 0 | 0 |  | 0 | 0 | 0 |  | 0 |  |  | 0 |
|  | Dentary, Meckel’s canal (0: open) [181] | **0** | 0 | 0 | 0 | 0 | 0 | 0 | 0 | 0 | 0 | 0 | 0 | 0 | 0 |  | 0 |
|  | Splenial, extent of anteromedial walling of Meckel’s canal (0: less than ½; 1: more than ^2^/_3_ of the dentary) [189] | **1** | 1 | 1 | 0 | 1 | 1 | 0 | 0 | 1 | 0 | 1 | 0 | 0 | 0 |  | 1 |
|  | Articular, retroarticular (0: posteriorly directed/ 1: medially deflected) [205] | **1** | 1 | 1 | 1 | 0 | 1 | 1 |  | 1 | 1 | 1 |  |  | 1 | 0 | 1 |
|  | Articular, retroarticular process with posterior broadening (0: abs/1: pre) [207] | **0** | 0 | 1 |  | 0 |  | 0 |  | 0 | 0 | 0 |  |  | 0 | 0 | 0 |
|  | Articular, tubercle on medial margin of retroarticular process (0: abs/1: pre) [209] | **0** | 0 | 0 |  | 0 | 1 | 0 |  | 0 | 0 | 0 |  |  | 0 |  | 0 |
|  | Dentition, posterior marginal tooth implantation (0: labially pleurodont/ 2: modified pleurodont) [214] | **{02}^a^** | 0 | 0 | 2 | 0 | 0 | 2 |  | 2 | 2 | 2 | 2 | 0 | 2 |  | 2 |
|  | Interclavicle, lateral arms (0: pre/1: abs)[270] | **0** | 0 | 0 |  | 0 | 0 | 1 |  | 0 | 0 | 0 |  |  |  |  | 0 |
| Platynota affinities | Maxilla, apex of nasal process relative to midpoint of maxilla (0: at or anterior to/1: posterior to) [28] | **1** | 0 | 0 | 0 | 0 | 0 | 0 | 0 | 1 | 1 | 1 | 0 | 1 | 1 | 0 | 1 |
|  | Maxilla, posterior extent of tooth row compared to anterior border of orbit (0: beyond/1: terminates at) [33] | **1** | 0 | 0 | 0 | 0 | 0 | 1 |  | 1 | 1 | 1 |  | 1 | 1 | 0 | 1 |
|  | Mandible, intramandibular septum posterior extent (0: more than two tooth widths anterior to/1: terminates at last tooth position) [170] | **1** | 0 | 0 | 0 | 1 |  | 1 | 1 | 1 | 1 | 1 | 1 | 1 | 1 |  | 1 |
|  | Dentary, subdental shelf (0: pre/1: abs) [182] | **1** | 1 | 1 | - | 0 |  | 1 | 1 | 1 | 1 | 1 |  | 0 |  |  | 1 |
|  | Splenial, posterior extent to coronoid apex (0: posterior to/1: at or anterior to) [190] | **1** | 0 | 0 | 1 | 1 | 0 | 1 |  | 1 | {01} | 1 |  | 1 | 1 |  | 1 |
|  | Coronoid, long and low anterior process (0: abs/1: pre) [196] | **1** | 0 | 0 | 1 | 0 | 0 | 1 |  | 1 | 1 | 1 |  | 1 | 1 |  | 1 |
|  | Surangular, anterior border when disarticulated (0: tapering/1: expanded anterodorsally) [200] | **1** | 0 | 0 | 0 | 0 | 0 | 1 |  | 1 | 1 | 1 |  |  | 1 |  | 1 |
|  | Dentition, form of teeth(0: straight, pointed; 2: trenchant, curved; 3: incipient cusps on posterior teeth) [212] | **2** | 3 | 0 | 0 | 2 | 2 | 2 | 2 | 2 | 2 | 2 | 2 | 2 | 2 | 2 | 2 |
|  | Dentition, spacing (0: close/1: wide) [211] | **1** | 0 | 0 | 0 | 0 | 0 | 1 | 1 | 1 | 1 | 1 | 1 | 1 | 1 | 1 | 1 |
|  | Dentition, expanded bases on marginal teeth (0: abs/1: pre) [217] | **1** | 0 | 0 | 0 | 1 |  | 1 | 1 | 1 | 1 | 1 | 1 | 1 | 1 |  | 1 |
|  | Dentition, chisel shaped posterior teeth (0: abs/1: pre) [226] | **0** | 0 | 1 | 0 | 0 | 0 | 0 | 0 | 0 | 0 | 0 | 0 | 0 | 0 | 0 | 0 |
|  | Vertebrae, precondylar constriction (0: absent; 1: weak; 2: strong) [233] | **1** | 0 | 0 |  | 0 | 0 | 0 | 0 | 1 | 1 | 2 | 1 |  |  | 1 | 0 |
|  | Ilium, anterior process (0: pre/1: abs) [287] | **0** | 1 | 1 |  | 1 | 0 | 1 |  | 0 | 1 | 0 |  |  |  |  | 1 |
| Conflicting with Platynota affinities | Jugal, posteroventral process (0:pre/1:abs) [48] | **0** | 0 | 0 |  | 0 | 0 | 0 |  | 1 | 1 | 1 |  | 1 | 1 | 0 | 1 |
|  | Vomer, shape (0: platelike/1: rod-like) [107] | **0** | 0 | 0 |  | 0 |  | 1 |  | 1 | 0 | 1 |  | 0 | 1 |  | 1 |
|  | Pterygoid, teeth (0:multiple rows; 1: single line; 2: absent) [118] | **2** | 2 | 0 | 0 | 1 | 0 | 2 |  | 2 | 1 | 2 | 1 | 0 | 0 |  | 1 |
|  | Parabasisphenoid, posterolateral flanges laterally overlying basioccipital (0: abs/1: pre) [143] | **1** | 1 | 1 | 1 | 1 | 1 | 1 |  | 0 | {01} | 0 |  |  | 1 |  | 1 |
|  | Parabasisphenoid, relationship with the posterior opening of the Vidian canal (0: houses it; 1: share it with the prootic)[146] | **1** | 1 | 1 | 1 | 1 |  | 1 |  | 1 | 1 | 1 |  | 0 |  |  | 0 |
|  | Mandible, external border of the anterior surangular foramen (0: formed only by the surangular; 1: margin with coronoid contribution; 3: with coronoid and dentary contribution) [173] | **0** | 3 | 0 | 0 | 0 | 0 | 0 |  | 1 | 1 | 1 |  |  |  |  | 0 |
|  | Dentary, coronoid and surangular processes(0: absent; 1: present) [185] | **1** | 1 | 1 | 1 | 1 | 1 | 0 |  | 0 | 0 | 0 |  | 1 | 0 |  | {-0} |
|  | Dentary, posterior extend of angular process compared to surangular process (0: same level; 1: anteriorly; 2: posteriorly) [186] | **0** | 0 | 1 | 0 | {02} | 2 | - |  | {-2} | - | - |  | 2 | - |  | - |
|  | Coronoid, posterior extent of the labial flange (0: abs; 1: labially, moderate; 2: labially beyond coronoid process) [193] | **0** | {12} | 1 | 1 | 1 | 1 | 0 |  | 1 | 0 | 1 |  | 1 | 1 |  | 1 |
|  | Coronoid, anterior end (0: clasps the dentary/1: butts against dentary) [194] | **1** | 0 | 0 | 0 | 0 | 0 | 1 |  | 0 | {01} | 0 |  | 1 | 1 |  | 1 |
|  | Interclavicle, anterior process (0: abs; 1: single; 2: double) [269] | **1** | 0 | 1 |  | 1 | 1 | - |  | 0 | 2 | 0 |  |  |  |  | 1 |
| Autapomorphies | Dentary, anterior inferior alveolar foramen (0: does not contribute; 1: contributes to dorsal border; 3: indistinct) [183] | **0** | 1 | 1 |  | {01} |  | 1 | 0 | 1 | 1 | 1 | 0 | 1 | 1 |  | 3 |
|  | Caudal vertebrae, autotomy planes (0: present on transverse process(es); 1: present posterior to the transverse process(es); 2: abs) [252] | **1** | 2 | 0 |  | 0 | 0 | 2 |  | 2 | 2 | 2 | 2 |  |  | 2 | 2 |

Columns represent states of character for the Phu Phok embryos and taxa amongst anguimorph. For taxa preceded by the mention ‘mode’, the state of character utilised is the mode (i.e., most common value) from the matrix of Conrad et al. (2011). When two states of character were equally distributed, both states are represented inside brackets. Rows describe the different states for the considered characters (numbers inside bracket indicate the character number utilised by Conrad et al. 2011). Characters are selected and sorted to highlight phylogenetic affinities of the embryos from Phu Phok. Abbreviations: A., Anguidae; Angui., Anguioidea; C., Carusioidea; M., Mosasauriformes; Monst. Monstersauria; Shini., Shinisauria

^a^The developmental stage of the Phu Phok embryos does not allow to define clearly the type of tooth implantation
